# Comparison of the Value of Color Doppler Ultrasound and Multislice Spiral CT in the Differential Diagnosis of Benign and Malignant Nodules in the Liver

**DOI:** 10.1155/2022/5251966

**Published:** 2022-02-02

**Authors:** Peiwan Jia, Ruirong Liu, Yun Liu, Yanyan Wu, Ting Dou

**Affiliations:** ^1^Department of Imaging, Jiaozhou Central Hospital, Qingdao 266300, China; ^2^Department of Ultrasound, Qingdao Xinkang Hospital, Qingdao 266000, China

## Abstract

**Objective:**

This study aimed to explore the value of color Doppler ultrasound and multislice spiral CT (MSCT) in the differential diagnosis of benign and malignant nodules in the liver.

**Methods:**

The clinical imaging data of 102 patients with nodular hepatocellular carcinoma (hepatocellular carcinoma group) and 50 patients with focal nodular hyperplasia (FNH) of the liver (FNH group) admitted to our hospital were collected, and their color Doppler ultrasound and MSCT imaging features were retrospectively analyzed to explore the value of their clinical application in the differential diagnosis of benign and malignant nodules in the liver.

**Results:**

The sensitivity, accuracy, and negative predictive value of MSCT in the diagnosis of nodular liver cancer were 94.12%, 92.76%, and 88.24%, respectively, which were significantly higher than those of color Doppler ultrasound 79.41%, 84.21%, and 69.12%, and the difference was statistically significant (*P* < 0.05).

**Conclusion:**

In conclusion, the value of MSCT in the differential diagnosis of benign and malignant liver nodules was significantly better than color Doppler ultrasound.

## 1. Introduction

Nodular hepatocellular carcinoma and focal nodular hyperplasia (FNH) are common types of hepatic solid occupancies, which are malignant and benign lesions of the liver, respectively [1, 2], and their diagnosis and differential diagnosis are the basis for rational diagnostic and therapeutic measures [3]. The pathogenesis of nodular hepatocellular carcinoma and focal nodular hyperplasia of the liver cannot be fully explained clinically, and because both lesions are liver lesions with abundant blood supply, the morphological characteristics of the two diseases have certain similarities and morphological features have certain overlap, which make the differential diagnosis of some lesions difficult and increase the difficulty of clinical diagnosis and treatment, making the clinical differential diagnosis more difficult [4–6]. Color Doppler ultrasonography is the most commonly used imaging method for liver diseases, which can localize and qualitatively diagnose lesions, but the diagnostic value is limited due to the poor resolution of the image and the susceptibility to external factors [7, 8]. Multislice spiral CT (MSCT) has various advantages such as high spatial resolution, large temporal resolution, noninvasive, and high efficiency, and it can also display small lesions more clearly, which can provide an important basis for the clinical diagnosis of liver nodules [9–11].

In this study, color Doppler ultrasound and MSCT were used to differentiate nodular hepatocellular carcinoma from focal nodular hyperplasia in the liver and to compare their clinical value in the differential diagnosis of benign and malignant nodules in the liver. We hypothesized that MSCT was more sensitive than color Doppler ultrasound in differentiating nodular hepatocellular carcinoma from focal nodular hyperplasia in the liver.

## 2. Materials and Methods

### 2.1. Clinical Data

**Figure 1:**
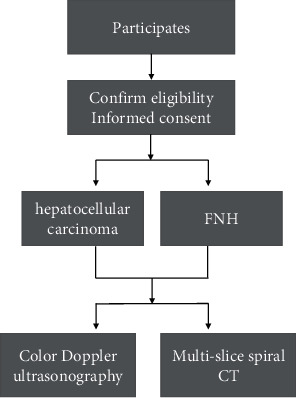
Flow diagram of participants in our study.

Clinical imaging data of 102 patients with nodular hepatocellular carcinoma (hepatocellular carcinoma group) and 50 patients with focal nodular hyperplasia of the liver (FNH) (benign group) admitted to our hospital from January 2015 to December 2018 were collected and retrospectively analyzed, and all patients underwent color Doppler ultrasound and MSCT before treatment. The study was approved by the Ethics Committee of Jiaozhou Central Hospital, and patients signed an informed consent form to participate in the study on a voluntary basis.  Figure 1 shows the flow of patients through the study.

### 2.2. Inclusion and Exclusion Criteria

#### 2.2.1. Inclusion Criteria

The inclusion criteria were as follows: the liver was found to be an occupying lesion with a single nodular lesion on imaging; all were confirmed to be nodular hepatocellular carcinoma or FNH by histopathological examination; it was the first visit and no radiotherapy treatment was administered before the visit; and the clinical and imaging data were complete.

#### 2.2.2. Exclusion Criteria

The exclusion criteria were as follows: patients with other types of liver space-occupying lesions; patients with metastatic liver cancer; patients with acute infectious diseases and congenital liver malformations; patients with severe liver and kidney dysfunction; patients who could not cooperate with the study due to various reasons; and patients with Incomplete research data.

## 3. Methods

### 3.1. Color Doppler Ultrasonography

The patient was placed in a supine position with a morning fast of more than 8 hours, and each section of the liver was carefully scanned by a physician specialized in ultrasound. The liver parenchyma, the bile duct, and portal venous system were observed in a left-to-right, top-down sequence, and the lesion site, morphology, margins, echogenicity, and relationship with adjacent tissues were observed and analyzed. Subsequently, the blood supply and blood flow within and around the lesion were observed by color Doppler ultrasound mode. The blood flow signal was graded as [12] follows: grade 0 was no blood supply within the tumor; grade 1 had less blood supply within the tumor, 1-2 punctate blood flows; grade 2 had abundant blood flow in the tumor, usually 3-4 punctate blood flow or 1-2 vessels; grade 3 has abundant blood flow in the tumor, more than 4 punctate blood flow or more than 2 vessels. The blood flow parameters such as hepatic artery diameter (mm), peak hepatic artery flow velocity (cm/s), minimum hepatic artery flow velocity (cm/s), portal vein flow velocity (cm/s), and resistance index (RI) were also measured with the help of PW mode. Each patient was measured three times, and the average value was taken.

### 3.2. Multislice Spiral CT Examination

The patient was examined in the supine position, and an axial and oblique scan was performed from the top of the diaphragm to the iliac crest, while a thin-layer scan of the liver region was performed. Conventional scan parameters: layer thickness 10 mm, layer spacing 10 mm, tube voltage 120 kV, tube current 120 mAs, scan matrix 512×512, layer thickness changed to 5 mm after the discovery of the lesion, other parameters remained unchanged, and the lesion was scanned plainly. Enhancement scan: iohexol (Bayer Pharma, State Drug Administration H10970416) was injected at a rate of 3 m/s using a syringe at a dose of 80–100 mL. The dynamic phase scan was performed within 10–30 s after iohexol injection using the intraabdominal MSCT value monitoring technique at the abdominal stem level, and the scan was delayed for 8 s after reaching the threshold of 100 HU, and the portal vein phase scan was performed 30 s after the end. Two sets of reconstructions were performed, one with conventional mixed energy images and the other with 70 keV single energy, and imported into the energy spectrum analysis software for analysis.

### 3.3. Statistical Analysis

The data were analyzed by SPSS 21.0 software and expressed by the mean ± standard deviation (‾*x* ± *s*). The comparison between groups was performed by the *t*-test; the count data were expressed by the rate (%), and the chi-square test was used. The value of color Doppler and MSCT in the diagnosis of nodular liver cancer was statistically calculated by the four-grid table method, and the difference was statistically significant with *P* < 0.05.

## 4. Results

### 4.1. Comparison of Color Doppler Ultrasound Characteristics of the Liver in the Nodular Hepatocellular Carcinoma Group and the FNH Group

**Figure 2:**
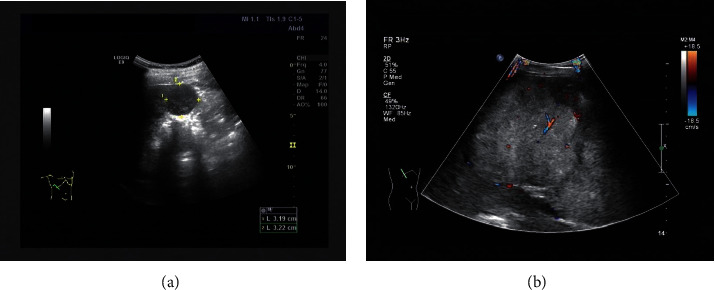
The color Doppler ultrasound manifestation of hepatocellular carcinoma. (a) The patient was a 58-year-old male with a history of hepatitis B. Two-dimensional ultrasound of the liver shows that 3.2*∗*3.2 cm hypoechoic nodules were visible in the liver, and the boundary was still clear. (b) Blood flow signals can be seen inside and around the mass. Pulse Doppler showed that the blood flow was high speed and high impedance, RI: 0.83.

Clinical imaging data of 102 patients with nodular hepatocellular carcinoma (hepatocellular carcinoma group) and 50 patients with focal nodular hyperplasia of the liver (FNH) (benign group) admitted to our hospital from January 2015 to December 2018 were collected and retrospectively analyzed, and all patients underwent color Doppler ultrasound and MSCT before treatment. Hepatocellular carcinoma group: 69 males and 33 females, aged 35–82 years, mean (59.23 ± 12.35) years; FNH group: 35 males and 15 females, age 36–85 years, mean (58.29 ± 12.67) years. The differences in gender and age between the two groups were not statistically significant (*P* > 0.05) and were comparable (Table 1). The color Doppler ultrasound manifestation of hepatocellular carcinoma was that the interior of the lesion was isoechoic, hypoechoic, and hyperechoic. The tumor had no obvious capsule and irregular margins, and the lesion was rich in blood flow signal and showed “high speed and high resistance” (Figure 2). The incidence of portal vein cancer thrombosis, cirrhosis, and lymph node enlargement was higher in the nodular hepatocellular carcinoma group than in the FNH group, the incidence of central scar in nodular hepatocellular carcinoma group was lower than that in the FNH group, and the proportion of lesions located under the liver capsule in the nodular hepatocellular carcinoma group was lower than in the FNH group, and the differences were statistically significant (*P* < 0.05) (Table 2).

### 4.2. Comparison of Color Doppler Ultrasound Blood Flow Parameters between the Nodular Hepatocellular Carcinoma Group and FNH Group

As given in Table 3, the hepatic artery diameter, peak flow velocity, minimum flow velocity, and resistance index (RI) were significantly higher in the nodular hepatocellular carcinoma group than in the FNH group (*P* < 0.05), and the portal vein flow velocity was significantly lower than in the FNH group (*P* < 0.05).

### 4.3. Comparison of Blood Flow Signals in Patients in the Nodular Hepatocellular Carcinoma Group and FNH Group

Nodular hepatocellular carcinoma showed mainly grades 2-3 blood flow signal, while the FNH group showed mainly grades 0-1. The difference in blood flow signal grading between the two groups was statistically significant (*P* < 0.01) (Table 4).

### 4.4. Comparison of MSCT Images of Nodular Hepatocellular Carcinoma and FNH

**Figure 3:**
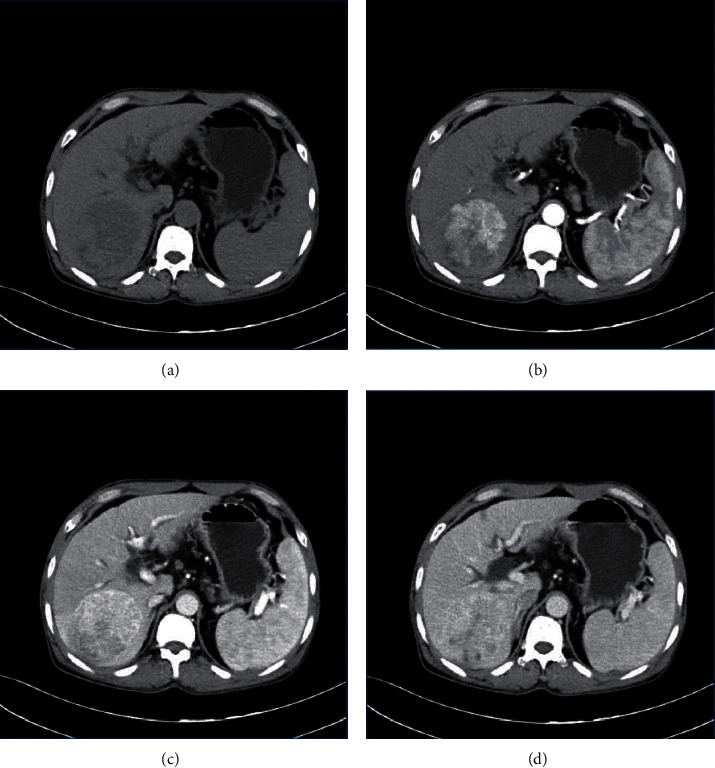
MSCT image of hepatocellular carcinoma. Male patient, 62 years old.

**Figure 4:**
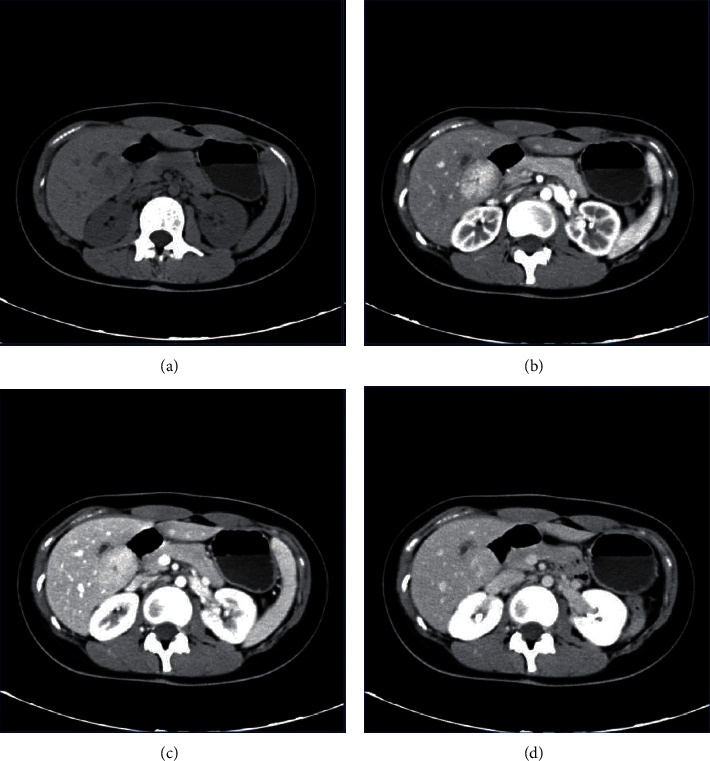
MSCT image of focal nodular hyperplasia of liver. Female patient, 52 years old.

The MSCT scan of nodular hepatocellular carcinoma lesions was predominantly low density, with a few showing calcifications. Nodular hepatocellular carcinoma showed “fast revealing and fast out” intensification with heterogeneous intensification, including patchy and nodular intensification in the arterial phase scan, and the degree of intensification in the venous and equilibrium phases continued to decrease (Figure 3). In MSCT scan or multiphase enhancement scan of focal nodular hyperplasia of the liver, the lesions have uniform internal density, and low-density scar structures can be seen in the center of large-sized lesions. Significant enhancement could be observed in the arterial phase on enhancement scan, and the enhancement of lesions in the venous and delayed phases decreased (Figure 4).

### 4.5. Comparison of Quantitative Parameters of MSCT Energy Spectrum between the Nodular Hepatocellular Carcinoma Group and FNH Group

The standardized iodine concentration and concentration of iodine in the lesion and the surrounding liver tissue were lower in arterial and portal stage nodular hepatocellular carcinoma than in focal nodular hyperplasia of the liver (*P* < 0.01) (Table 5).

### 4.6. Comparison of Noise Ratio at Different Energy Levels in MSCT of Patients with Nodular Hepatocellular Carcinoma and FNH

The noise ratios of hepatocellular carcinoma at 40 keV, 90 keV, and 140 keV energy levels in the arterial phase were all lower than those of the FNH group, and the differences were statistically significant (*P* < 0.01). The noise ratios of hepatocellular carcinoma at 90 keV and 140 keV energy levels in the venous phase were all higher than those of the FNH group, and the differences were statistically significant (*P* < 0.01) (Table 6).

### 4.7. Comparison of Diagnostic Value of Color Doppler Ultrasound and Multilayer Spiral MSCT in Nodular Hepatocellular Carcinoma

Using pathological diagnosis as the gold standard, the sensitivity, accuracy, and negative predictive value of MSCT for the diagnosis of hepatocellular carcinoma were significantly improved compared with color Doppler ultrasound (*P* < 0.05) (Table 7).

## 5. Discussion

Primary hepatocellular carcinoma is currently the sixth most common cancer in the world, ranking fourth in cancer-related deaths and posing a serious threat to human health [13]. Since early symptoms of hepatocellular carcinoma are nonspecific, patients are often at advanced stages when they come to hospital and the prognosis is poor, so early diagnosis is especially important [14, 15]. The effective treatment for early stage hepatocellular carcinoma is surgical resection and liver transplantation, and about 85% of patients with advanced hepatocellular carcinoma lose the opportunity of surgical treatment [16, 17]. Both nodular hepatocellular carcinoma and focal nodular hyperplasia of the liver present as occupying lesions of the liver, and the clinical manifestations and morphological features of both have some overlaps, which are not easy to differentially diagnose in early stages [18, 19]. Therefore, it is extremely important to use effective measures for the differential diagnosis of nodules in a timely manner. Imaging tools such as color Doppler ultrasound and MSCT are often used clinically to differentially diagnose nodular hepatocellular carcinoma from focal hyperplastic lesions of the liver.

Color Doppler ultrasonography has the advantages of easy operation, low cost, and high tissue resolution, making it the preferred imaging method for occupying liver lesions. The results of this study showed that the benign and malignant lesions could also be identified by changes in blood flow signal within the lesion. However, color Doppler ultrasonography is easily influenced by factors such as respiration, body position, obesity, acoustic window, and angle, which can lead to a high rate of missed diagnosis and misdiagnosis, especially in the context of cirrhosis, where the echogenic characteristics of small hepatocellular carcinoma and benign hepatic hyperplastic nodules are more similar, and the lack of envelope or acoustic corona nearby leads to a high rate of misdiagnosis and difficulty in characterization [20]. Therefore, the differential diagnosis of benign and malignant hepatic nodules by color Doppler ultrasound alone is of limited value.

Compared with ultrasound, MSCT is not interfered by obesity, breathing, and other factors and can clearly display the anatomical structure of the human liver, with various advantages such as high spatial resolution, large temporal resolution, noninvasive, and high efficiency. MSCT can also display small lesions more clearly, which can provide an important basis for clinical diagnosis. In the reconstruction process, the use of different reconstruction modes can provide more information and better observation angles [21]. The MSCT scan of primary nodular hepatocellular carcinoma lesions is predominantly low density, with a few calcifications; it shows “fast revealing and fast out” intensification, with uneven intensification, and the arterial phase scan shows patchy and nodular intensification, and the venous and equilibrium phases show a continuous decrease in intensification [22, 23]. The MSCT scan of focal nodular hyperplasia in the liver is predominantly slightly lower density, with heterogeneous density and stellate, striated, or fissured, and most of the visible lesions have typical central scars, which have a high differential value [24, 25]. However, there is a certain percentage of misdiagnosis due to the overlap between the imaging manifestations of the two. In this study, the analysis of reconstructed images showed that the standardized iodine concentration, lesion, and surrounding liver tissue iodine were higher for focal nodular hyperplasia of the liver in the arterial and portal phases compared with nodular hepatocellular carcinoma. Moreover, the noise ratio was higher for hepatocellular carcinoma at different energy levels in the arterial phase and lower for venous. It is suggested that the difference in iodine concentration and noise ratio between nodular hepatocellular carcinoma and focal nodular hyperplasia of the liver after reconstruction by images was significant and could be used as a basis for differentiating between the two.

In conclusion, color Doppler ultrasound could diagnose nodular hepatocellular carcinoma and focal lesions of the liver effectively by two-dimensional ultrasound and blood flow at the lesion site, and MSCT could effectively diagnose nodular hepatocellular carcinoma and focal lesions of the liver by different indicators such as enhancement and quantification of portal vein and arterial phase energy spectrum. MSCT was of a higher diagnostic value in identifying benign and malignant nodules in the liver. Nevertheless, the results should be further confirmed in a larger number of patients in the future study.

## Figures and Tables

**Table 1:** Baseline comparison between the two groups.

Variable	Hepatocellular carcinoma (*n* = 102)	FNH group (*n* = 50)	*P*
Gender			0.769
Male	69	35	
Female	33	15	

Age (years)	59.23 ± 12.35	58.29 ± 12.67	0.412

Cirrhosis			0.730
Yes	52	24	
No	50	26	

HBV			0.923
Negative	60	29	
Positive	42	21	

**Table 2:** Comparison of color Doppler ultrasound characteristics of the liver in the nodular hepatocellular carcinoma group and the FNH group (*n* (%)).

Group	*n*	The lesion located under the liver capsule	Central scar	Portal vein tumor thrombus	Liver cirrhosis	Swollen lymph nodes
Nodular hepatocellular carcinoma	102	22 (21.57)^a^	15 (14.71)^a^	22 (21.57)^a^	61 (59.80)^a^	20 (18.18)^a^
FNH	50	26 (52.00)	31 (62.00)	0 (0.00)	7 (14.00)	1(2.00)
*X* ^2^		14.381	35.560	12.609	28.473	13.760
*P*		≤0.001	≤0.001	≤0.001	≤0.001	≤0.001

Compared with the FNH group, ^a^*P*<0.01.

**Table 3:** Comparison of color Doppler ultrasound blood flow parameters between the nodular hepatocellular carcinoma group and FNH group (‾*x* ± *s*).

Group	*n*	Hepatic artery diameter (mm)	Peak hepatic artery velocity (cm/s)	Minimum flow velocity of hepatic artery (cm/s)	Resistance index (RI)	Portal vein flow rate (cm/s)
Nodular hepatocellular carcinoma	102	5.56 ± 0.42^b^	118.32 ± 15.73^b^	22.65 ± 3.12^b^	0.84 ± 0.06^b^	10.52 ± 1.08^b^
FNH	50	4.02 ± 0.31	63.25 ± 5.97	8.69 ± 1.62	0.44 ± 0.03	18.01 ± 3.69
*t*		3.310	7.963	3.569	5.301	-3.126
*P*		0.015	≤0.001	0.012	0.001	0.029

Compared with the FNH group, ^b^*P* < 0.05.

**Table 4:** Comparison of blood flow signals in patients in the nodular hepatocellular carcinoma group and FNH group (*n* (%)).

Group	*n*	Grade 0	Grade 1	Grade 2	Grade 3
Nodular hepatocellular carcinoma	102	0 (0.00)^c^	13 (12.75)^c^	53 (51.96)^c^	36 (35.29)^c^
FNH	50	30 (60.00)	18 (36.00)	2 (4.00)	0 (0.00)
*X* ^2^		76.249	11.176	33.423	23.124
*P*		≤0.001	≤0.001	≤0.001	≤0.001

Compared with the FNH group, ^c^*P* < 0.01.

**Table 5:** Comparison of quantitative parameters of MSCT energy spectrum between the nodular hepatocellular carcinoma group and FNH group ($\overset{¯}{x}$ ± *s*).

Group	*n*	Standardized iodine concentration		Iodine concentration in the lesion and surrounding liver tissue	
		Arterial phase	Portal phase	Arterial phase	Portal phase
Nodular hepatocellular carcinoma	102	0.21 ± 0.10^d^	0.51 ± 0.15^d^	3.15 ± 0.79^d^	0.93 ± 0.16^d^
FNH	50	0.46 ± 0.19	0.94 ± 0.32	5.97 ± 1.68	1.31 ± 0.29
*t*		10.235	13.264	16.389	8.674
*P*		≤0.001	≤0.001	≤0.001	≤0.001

Compared with the FNH group, ^d^*P* < 0.01.

**Table 6:** Comparison of noise ratio at different energy levels in MSCT of patients with nodular hepatocellular carcinoma and FNH ($\overset{¯}{x}$ ± *s*).

Group	*n*	Arterial phase			Portal phase		
		40 KeV	90 KeV	140 KeV	40 KeV	90 KeV	140 KeV
Nodular hepatocellular carcinoma	102	3.21 ± 1.18^e^	0.73 ± 0.35^e^	0.31 ± 0.08^e^	1.10 ± 0.19	1.23 ± 0.53^e^	0.84 ± 0.23^e^
FNH	50	7.69 ± 1.53	2.20 ± 0.79	0.57 ± 0.15	1.09 ± 0.21	0.32 ± 0.11	0.45 ± 0.12
*t*		16.325	18.452	10.324	0.408	9.374	11.234
*P*		≤0.001	≤0.001	≤0.001	0.685	≤0.001	≤0.001

Compared with the FNH group, ^e^*P* < 0.01.

**Table 7:** Comparison of the diagnostic value of color Doppler ultrasound and MSCT in nodular hepatocellular carcinoma (% (*n*)).

Detection indicator	Sensitivity	Specificity	Accuracy	Positive predictive value	Negative predictive value
Color Doppler ultrasound	79.41 (81/102)	94.00 (47/50)	84.21 (128/152)	96.43 (81/84)	69.12 (47/68)
MSCT	94.12 (96/102)^f^	90.00 (45/50)	92.76 (141/152)^f^	95.05 (96/101)	88.24 (45/51)^f^
*X* ^2^	9.605	0.543	5.457	0.211	7.587
*P*	0.002	0.461	0.019	0.646	0.006

Compared with the FNH group, ^f^*P* < 0.01.

## Data Availability

The data used to support the findings of this study are available from the corresponding author upon request.

## References

[1] Roncalli M., Sciarra A., Tommaso L. D. (2016 Jun). Benign hepatocellular nodules of healthy liver: focal nodular hyperplasia and hepatocellular adenoma. *Clinical and Molecular Hepatology*.

[2] Renzulli M., Pettinari I., Vasuri F. (2021). Liver collision lesion: inflammatory hepatocellular adenoma within focal nodular hyperplasia. *Gastroenterology Report*.

[3] Bilreiro C., Soler J. C., Ayuso J. R., Caseiro-Alves F., Ayuso C. (2021). Diagnostic value of morphological enhancement patterns in the hepatobiliary phase of gadoxetic acid-enhanced MRI to distinguish focal nodular hyperplasia from hepatocellular adenoma. *La radiologia medica*.

[4] Luo M., Zhang L., Jiang X. H., Zhang W. D. (Jul-Aug 2017). Intravoxel incoherent motion: application in differentiation of hepatocellular carcinoma and focal nodular hyperplasia. *Diagnostic and interventional radiology*.

[5] Li W., Li R., Zhao X. (2021). Differentiation of hepatocellular carcinoma from hepatic hemangioma and focal nodular hyperplasia using computed tomographic spectral imaging. *Journal of clinical and translational hepatology*.

[6] Roux M., Pigneur F., Baranes L. (2018). Differentiating focal nodular hyperplasia from hepatocellular adenoma: is hepatobiliary phase MRI (HBP-MRI) using linear gadolinium chelates always useful?. *Abdominal Radiology*.

[7] Iwasaki M., Furuse J., Yoshino M., Ryu M., Moriyama N., Mukai K. (Jul-Aug 1998). Sonographic appearances of small hepatic nodules without tumor stain on contrast-enhanced computed tomography and angiography. *Journal of Clinical Ultrasound*.

[8] Hui R., Li Z., Liu Z., Liu X., Deng H. (2021). The clinical value of color Doppler ultrasonography in measuring the hemodynamics of liver cirrhosis patients’ portal and splenic veins. *American Journal of Tourism Research*.

[9] Esser M., Schneeweiß S., Kolb M. (2017 Apr). Comparison between acoustic radiation force impulse quantification data and perfusion-CT parameters in hepatocellular carcinoma. *European Journal of Radiology*.

[10] Sun W. J., Gao Z. L., Gao Y. J. (2020). [Quantitative evaluation of early stage blood flow change status after radiofrequency ablation based on multi-slice spiral CT whole-liver perfusion imaging on small hepatocellular carcinoma]. *Zhonghua Gan Zang Bing Za Zhi*.

[11] Li M., Chen Y., Gao Z., Zhu K., Yin X. (2015). [Evaluation of the blood flow in common hepatic tumors by multi-slice spiral CT whole-liver perfusion imaging]. *Zhonghua Zhongliu Zazhi*.

[12] Lu L.-C., Shao Y.-Y., Chan S.-Y., Hsu C.-H. (2014 Feb). Ann-Lii Cheng. Clinical characteristics of advanced hepatocellular carcinoma patients with prolonged survival in the era of anti-angiogenic targeted-therapy. *Anticancer Research*.

[13] Siegel F. B., Ferlay J., Soerjomataram I., Siegel R. L., Torre L. A., Jemal A. (2018 Nov). Global cancer statistics 2018: GLOBOCAN estimates of incidence and mortality worldwide for 36 cancers in 185 countries. *CA: A Cancer Journal for Clinicians*.

[14] Meer S. V., de Man R. A., Siersema P. D., J van Erpecum K. (2013 Oct 28). Surveillance for hepatocellular carcinoma in chronic liver disease: evidence and controversies. *World Journal of Gastroenterology*.

[15] Tang B., Zhu J., Zhao Z. (2021). Diagnosis and prognosis models for hepatocellular carcinoma patient’s management based on tumor mutation burden. *Journal of Advanced Research*.

[16] Clark T., Maximin S., Meier J., Pokharel S., Bhargava P. (2015). Hepatocellular carcinoma: review of epidemiology, screening, imaging diagnosis, response assessment, and treatment. *Curr Probl Diagn Radiol. Nov-Dec*.

[17] Wu S.-Y., Liao P., Yan L.-Y. (2021). Correlation of MKI67 with prognosis, immune infiltration, and T cell exhaustion in hepatocellular carcinoma. *BMC Gastroenterology*.

[18] Sano K., Ichikawa T., Motosugi U. (2017 Feb). Outcome of hypovascular hepatic nodules with positive uptake of gadoxetic acid in patients with cirrhosis. *European Radiology*.

[19] Kitao A., Matsui O., Yoneda N. (2018). Differentiation between hepatocellular carcinoma showing hyperintensity on the hepatobiliary phase of gadoxetic acid-enhanced MRI and focal nodular hyperplasia by CT and MRI. *American Journal of Roentgenology*.

[20] Lee S. J., Won H. J., Kim K. W., Shin Y. M., Kim P. N. (2017 Sep). Value of contrast-enhanced sonography of small hepatocellular carcinoma with sonazoid prior to radiofrequency ablation. *Journal of Clinical Ultrasound*.

[21] Guo Y., Zhang Y., Huang J. (2016 Apr). Safety and efficacy of transarterial chemoembolization combined with CT-guided radiofrequency ablation for hepatocellular carcinoma adjacent to the hepatic hilum within milan criteria. *Journal of Vascular and Interventional Radiology*.

[22] Liu F.-Y., Li X., Yuan H.-J., Guan Y., Wang M.-Q. (2018 Oct 20). Angio-Computed tomograph-guided immediate lipiodol computed tomograph for diagnosis of small hepatocellular carcinoma lesions during transarterial chemoembolization. *Chinese Medical Journal*.

[23] Long X., Cao J., Shi L., Li W., Liu H. (2009). [MSCT perfusion imaging and its correlation with perfusion parameters, survivin expression, MVD, and pathologic grade in hepatocellular carcinomas]. *Zhong Nan Da Xue Xue Bao Yi Xue Ban*.

[24] Awai K., Takada K., Onishi H., Hori S. (2002). Aortic and hepatic enhancement and tumor-to-liver contrast: analysis of the effect of different concentrations of contrast material at multi-detector row helical CT. *Radiology*.

[25] Lee Y. J., Lee J. M., Lee J. S. (2015 Apr). Hepatocellular carcinoma: diagnostic performance of multidetector CT and MR imaging-a systematic review and meta-analysis. *Radiology*.

